# 

**Published:** 1836-01-01

**Authors:** 


### MEDICO-CHIRURGICAL

### REVIEW,

AND

### JOURNAL

OF

### PRACTICAL MEDICINE.

*(NEW SERIES.)*

VOLUME TWENTY-FOUR,

[1st of OCTOBER, 1835, to 31st of MARCH,]

1836.

VOL. IV. of DECENNIAL SERIES.

EDITED

BY JAMES JOHNSON, M.D.

PHYSICIAN EXTRAORDINARY TO THE KING,

AND

HENRY JAMES JOHNSON, ESQ.

LECTURER ON ANATOMY AT THE SCHOOL IN KINNERTON STREET, AND FORMERLY HOUSE SURGEON TO ST. GEORGE'S AND THE LOCK HOSPITALS.

Surgeon Gen'l's Office LIBRARY Washington D.C.

57464

LONDON:

PUBLISHED BY S. HIGHLEY, 32, FLEET STREET,

*Re-printed in New York, by Mr. Wood.*

1836.

WI
ME 75 T

PRINTED BY G. HAYDEN,
Little College Street, Westminster.

### BIBLIOGRAPHICAL RECORD;

OR,

*Works received for Review since last Quarter.*

1. Manual of Pathology, &c. By L. Martinet, M.D. Translated from the French, by Jones Quain, M.D. with Alterations and Additions. Fourth Edition, revised and enlarged, 12mo, pp. 443, price 7s. 6d. Sept. 1835.

☞ *This edition of a most valuable pocket companion, is very much enlarged and improved.*

---

2. A Treatise on the Functional and Structural Changes of the Liver, in the Progress of Disease, and on the Agency of Hepatic Derangement in producing other Disorders; with numerous Cases, exhibiting the Invasion, Symptoms, Progress, and Treatment of Hepatic Disease in India. By W. E. E. Conwell, M.R.I.A. Surgeon of the Madras Establishment, &c. Octavo, pp. 531. James Duncan, Paternoster-row, Sept. 1835.

3. A Series of Botanical Tables, and Tables of the Materia Medica, designed for the Use of Students preparing for Examination at Apothecaries' Hall. Illustrated with numerous Engravings on Wood, and four coloured Medico-botanical Maps of Europe, Asia, Africa, and America, shewing the Geographical Situation of all the Plants of the Pharmacopœia. By W. K. Toase, F.L.S. Lecturer on Botany and Anatomy. Quarto, Longman and Co. 1835. Price 4s.

---

4. The Anatomy of the Regions interested in the Surgical Operations performed upon the Human Body; with occasional Views of the Pathological Conditions which render the Interference of the Surgeon necessary. In a Series of Plates engraved on India Paper, the Size of Life. By J. Le-

BAUDY, M.D. with Additions. Quarto, Parts I., II., and III. Baillière, Sept. 1835. Price 1*l*. 4*s*.

---

5. A Popular Treatise on Diet and Regimen; intended as a Text-Book for the Invalid and the Dyspeptic. By W. H. ROBERTSON, M.D. Octavo, pp. 251. Chas. Tilt, Sept. 1835.

*A good Work.*

---

6. Outlines of Human Pathology. By HERBERT MAYO, F.R.S. Professor of Anatomy, &c. Octavo, pp. 264. Renshaw, Strand, Oct. 1835.

---

7. Remarks on the Theory and Treatment of Scarlet Fever; with brief Notices of the Disease, as it prevailed epidemically at Bridlington in 1831. By HUMPHREY SANDWITH, M.D. Octavo, pp, 48. Highley, Sept. 1835.

☞ *This is re-printed from the Edinburgh Journal, and is a very excellent monograph on an important subject.*

---

8. Observations on the principal Medical Institutions and Practice of France, Italy, and Germany; with Notices of the Universities, and Cases from Hospital Practice. To which is added, an Appendix on Animal Magnetism and Homœopathy. By EDWIN LEE, M.R.C.S. Octavo, pp. 216. Churchill, London, October, 1835.

---

9. An Introduction to Hospital Practice, in various Complaints; being a Clinical Report of Fever, Gout, Rheumatism, Cholera, Jaundice, Erysipelas, Insanity, &c. with Remarks on their Pathology and Treatment. By C. J. B. ALDIS, M.A. M.B. &c. 8vo, pp. 125. Longman & Co. Oct. 1835.

---

10. A Statistical Inquiry into the present State of the Medical Charities of Ireland; with Suggestions for a Medical Poor-Law, by which they may be rendered much more extensively efficient. By DENIS PHELAN, Surgeon to the Co. Tipperary Gaol, and to the House of Industry, &c. Octavo, pp. 325, with numerous Tables, &c. Oct. 1835.

---

11. A Treatise on the Causes and Cure of Stuttering, with reference to certain Modern Theories. By JAMES WRIGHT, Esq. late of Magdalen Hall, Oxford. Duodecimo, pp. 51. Whittaker, 1835.

☞ *Some useful hints may be gleaned by stammerers from this little treatise.*

12. Gregory's Conspectus. A Literal Interlinear Translation of the first ten Chapters of Gregory's Conspectus Medicinæ Theoreticæ, with the original Text. To which are added, the Ordo Verborum, and Rules for construing, &c. By ROBERT VENABLES, A.M. M.B. Oxon. Duodecimo, pp. 122. Sherwood and Co. Oct. 1835.

☞ *This is a capital grinder for the "Hall," at the trifling expence of four shillings and sixpence.*

---

13. Practical Anatomy of the Nerves and Vessels supplying the Head, Neck, and Chest; intended as a Guide for the Use of Students in the Dissection of those Structures. By EDWARD COCK, Demonstrator of Anatomy at Guy's Hospital. Small 8vo, pp. 242. Schloss, Great Russell-street, Bloomsbury, price 7s. Oct. 1835.

☞ *The portion of anatomy to which Mr. Cock has here directed his attention, is very difficult to learn or demonstrate. The author's plan of prosecuting the dissection of these intricate parts appears to be very judicious.*

---

14. Elements of Bed-side Medicine and General Pathology, or General Disease-discourse, with a Sketch of the Origin, Progress, and Prospects of Clinical Medicine and Surgery, &c. By J. STEWART THORBURN, M.D. Octavo, pp. 437. London, Longman and Co. Oct. 1835.

---

15. Practical Observations on Diseases of the Heart, Lungs, Stomach, Liver, &c. occasioned by Spinal Irritation; and on the Nervous System in general, as a Source of Organic Disease, illustrated by Cases. By JOHN MARSHALL, M.D. Octavo, pp. 172. London, Oct. 1835, price 6s. 6d.

---

16. North American Archives of Medical and Surgical Science. Edited by E. GEDDES, M.D. Professor of Anatomy and Physiology in the University of Maryland. Nos. I. to X. Published monthly.

---

17. An Introductory Address, delivered at the Commencement of the tenth Session of the Manchester School of Medicine and Surgery. By JAMES L. BARDSLEY, M.D.

---

18. On Bloodletting; an Account of the Curative Effects of the Abstraction of Blood. with Rules for employing both Local and General Bloodletting in the Treatment of Diseases. By JAS. WARDROP, M.D. Surg.

to the late King, &c. Small 8vo, pp. 148. Baillière, Oct. 1835, price 5s.

☞ *We reviewed this Lecture fully, in our Number for April,* 1834.

---

19. The Constitution of Man, considered in relation to External Objects. By Geo. Combe. 4th Ed. 1835.

---

20. Clinique Medicale; or Reports of Medical Cases. By G. Andral, M.D. Condensed and Translated by D. Spillan, M.D. Part 3, containing Diseases of the Chest. Octavo, pp. 200. Renshaw, Strand, Nov. 1835. Price *Five Shillings.*

☞ *This part is worth three—four times its price—containing the pathology of diseases of the chest. We recommend this important and cheap work to the library of every medical practitioner.*

---

21. The People's Edition of the Constitution of Man, considered in relation to External Objects. By George Combe. Fourth Edition, revised, corrected, and enlarged. Price 1s. 6d. Edinb. and London, Nov. 1835.

☞ *We need say nothing respecting a work that has gone through four large editions. This one is, indeed, given away, it is so cheap.*

---

22. On Dropsies, connected with Suppressed Perspiration and Coagulable Urine. By Jonathan Osborne, M.D. President of the King and Queen's College of Physicians, &c. Small 8vo, pp. 64. Sherwood and Co. 1835, price 5s. with a Coloured Plate.

---

23. A Treatise on Poisons, in relation to Medical Jurisprudence, Physiology, and the Practice of Physic. By Robert Christison, M.D. &c. Octavo, pp. 875. Third Edition. Edinburgh and London, Nov. 1835.

☞ *Although only four years have elapsed since the second edition, the author has been able to make considerable additions to, and introduce many improvements into, this edition. We need hardly say, that Dr. Christison's work is not merely the best, but the only classical book on toxicology in the English language. No practitioner is safe for one hour, without this or one of the preceding editions.*

---

24. St. Thomas's Hospital Reports. By John F. South, Assist.-Surgeon. No. I. Nov. 1835. pp. 118. Sherwood and Co. price 3s.

---

25. An Experimental Guide to Chemistry. By Edward Davy, M.R.C.S. 12mo, pp. 98, with Plates, price 3s. 6d. Printed for the Author, No. 390, Strand.

---

26. The Cyclopædia of Anatomy and Physiology. Edited by Robert B. Todd, M.B. Lecturer on Anatomy, &c. Part 2, Aug. 1835, containing—Animal Kingdom (Dr. Grant)—Animal (Dr. Willis)—Ankle (Dr. Brenan)—Ankle-Joint (Dr. Brenan)—Ankle-Joint, Abnormal Condition of (R. Adams, Esq.)—Annelida (Dr. Milne Edwards)—Auris (R. Harrison, Esq.)—Aorta (Dr. Hart)—Arachnida (Dr. Audouin.)

☞ *The work goes on with spirit, and increasing merit.*

---

27. The Principles and Practice of Obstetric Medicine, in a Series of Systematic Dissertations on Midwifery, and on the Diseases of Women and Children, illustrated by numerous Plates. By Dr. Davis. Part 43, pp. 44, and three Plates—Part 44, pp. 30, and one Plate, price *Sixpence!*

☞ *We are surprised how the publishers can afford such a quantity of letter-press, and large plates, for the sum of sixpence! This is now, without exception, the cheapest publication we have ever seen.*

---

28. An Inquiry, Physiological and Pathological, into the proximate Cause of Cholera. By Protheroe Smith, M.R.C.S. Senior Surgeon to the Farringdon Dispensary. Octavo, pp. 39. Bailliere, Nov. 1835.

☞ *An ingenious essay; but the proximate cause of cholera, and, indeed, of any disease, requires the sword of an Alexander to cut the Gordian knot.*

---

29. An address delivered at the first Anniversary Meeting of the Birmingham School of Medicine and Surgery. By Jas. Thos. Law, Chancellor of the Diocese of Lichfield.

☞ *This is an eloquent and classical address, inculcating on the minds of the students the necessity of combining correct morals, and an observance of religious obligations!! Our reverend Friend has paid many handsome compliments to the members of the medical profession—which we hope they deserve.*

30. The British Medical Almanac for 1836. To be continued annually. Small 8vo, pp. 160. Sherwood and Co. price Two Shillings.

☞ *This little volume contains a vast mass of information, extremely useful to the profession generally. The tabular views of diseases are very curious and instructive.*

---

31. Supplement to Outlines of Physiology. By W. P. Alison, M.D. &c. Octavo, pp. 73. Blackwood, Edinb. Nov. 1835.

*** *Meant to supply deficiencies and correct inferences.*

---

32. Observations on the Action of the Broom-seed in Dropsical Affections. By Richard Pearson, M.D. Octavo, pp. 15, with 3 Plates.

*** *The form of the tincture is two ounces of broom-seed, digested for eight days in eight ounces of proof-spirit. From one to three drachms may be taken thrice a-day. Dr. Eccles has used it successfully.*

---

33. A Manual of Practical Midwifery; containing a Description of Natural and Difficult Labours, with their Management. Intended chiefly as a Book of Reference for Students and Junior Practitioners. Illustrated by 15 Engravings. By James Reid, M.D. formerly House-Surgeon of the General Lying-in Hospital. Duodecimo, pp. 246, price 5s. 6d. Churchill, Dec. 1835.

☞ *If a* Vade Mecum *is at all allowable in the practice of medicine or surgery, it is in that department called Obstetricy, where one or two lives depend on the right step to be pursued, at the moment, and where the practitioner dare not leave the room of the patient to consult men or books.*

---

34. A Report on Small-pox, as it appeared in the Island of Ceylon, in 1833—34, with an Appendix. By J. J. Kinnis, M.D. 8vo, pp. 88, with plates, plans, &c. Colombo, 1825.

---

35. A new and complete Manual of Auscultation and Percussion, applied to the Diagnosis of Diseases. By M. A. Raciboroli, M.D. Paris. Translated by William Fitzherbert, B.A. Cambridge. Small 8vo, pp. 204. With a Synoptical Table, &c. London, A. H. Bailey and Co. Cornhill. Dec. 1835.

*** *We have no hesitation in saying, that this is decidedly the best, as well as most concentrated, Manual of Auscultation and Percussion in the English, or in any language.*

---

36. The Cyclopædia of Anatomy and Physiology. Part 4. Dec. 1835.

*** *The principal contributors are Drs. Benson, Hart, Harrison, Edwards, and Babington. Messrs. Owen, W. T. Brande, Phillips. This Part comes down to* "Blood," *morbid conditions of the. We have spoken of the work in another place.*

---

37. Lithographic Portrait of M. Tiedemann, Professor in the University of Heidelberg. Published by M. Schloss, 2, Great Russell-street.

*** *A striking likeness of this veteran and eminent physiologist.*

---

38. A Lecture introductory to the Study of Medical Science, delivered at the opening of the medical Classes of the Andersonian University, Session 1835-6. By Robert Hunter, M.D. Octavo, pp. 32.

---

39. A Practical Treatise on Widwifery, containing the result of sixteen thousand, six hundred and fifty-four Births, occurring in the Lying-in Hospital during the period of Seven Years, &c. By Robert Collins, M D. late Master of the Institution. Octavo, pp. 528. London, Longman and Co. 1835. *** *In our next.*

---

40. A Series of Anatomical Plates. By J. Quain, M.D. Fasciculi XXII. to XXX. price 2s. each. Division 2—Arteries 6.

*** *Most admirable Plates for the Anatomical Student.*

---

41 A Synoptical Chart of the Diseases of the Eye, classed according to their various Tissues, and the latest Nosological Arrangements, &c. By J. H. Curtis, Esq.

*** *This is a more scientific chart than its predecessor on the ear, which is saying a good deal for it. Much pains have been taken in the nosological arrangement.*

---

42. A Treatise on the Diseases of the Eye and its Appendages. By Richard Middlemore, M.R.C S. Surgeon to the Birmingham Eye Infirmary. In two Vols. pp. 1644. Longman and Co. Dec. 1835.

---

43. Rudiments of Physiology. By Dr. Fletcher. Part I. On Organism. 8vo, pp. 155. J. Caffræ, Edinb. Nov. 1835.

---

*Closed on the 18th Dec.*

